# A comprehensive review of heart failure: Unraveling the etiology, decoding pathophysiological mechanisms, navigating diagnostic modalities, exploring pharmacological interventions, advocating lifestyle modifications, and charting the horizon of emerging therapies in the complex landscape of chronic cardiac dysfunction

**DOI:** 10.1097/MD.0000000000036895

**Published:** 2024-01-19

**Authors:** Chukwuka Elendu, Dependable C. Amaechi, Tochi C. Elendu, Border-ere Fiemotonghan, Osinachi K. Okoye, Chigozirim M. Agu-Ben, Samuel O. Onyekweli, Dorcas A. Amapu, Richard Ikpegbu, Mercy Asekhauno, Erica Pius, Adediwura T. Bayo-Shodipo, Chidera A. Okezie-Okoye, Nurudeen Bello, Chibuike Oguine, Promise Edochie, Nkechinyere Dike, Ibirongbe Amos, Joan Asekhauno, Tanitoluwa M. Wusu-Ejalonibu, Emmanuella E. Ozigi, Grace O. Otobo, Aderinsola R. Olokodana, Chiagozie P. Ayabazu, Raphael T. Nwafor, Nandir J. Gonji, Otite Akpovona, Temitope I. Awotoye, Mutalib O. Ozigis, Oluwatosin Afolabi, Omotayo S. Alabi, Mololuwa Adebayo

**Affiliations:** a University of California, Santa Cruz, CA; b Igbinedion University, Okada, Nigeria; c Imo State University, Owerri, Nigeria; d Jackson State University, MS; e Chukwuemeka Odumegwu Ojukwu University Teaching Hospital, Awka, Nigeria; f Kharkiv National Medical University, Kharkiv, Ukraine; g Obafemi Awolowo University, Ife, Nigeria; h Babcock University, Ilishan-Remo, Nigeria; i King’s College Hospital NHS Foundation Trust, London, England; j Reddington Multispecialist Hospital, Lagos, Nigeria; k University of Ilorin Teaching Hospital, Ilorin, Nigeria; l King’s College Hospital NHS Foundation Trust, London, England; m University of Osun Teaching Hospital, Osogbo, Nigeria; n Ring Road State Hospital, Ibadan, Nigeria.

**Keywords:** diagnostic modalities, emerging therapies, etiology, heart failure, lifestyle modifications, pathophysiological mechanisms, pharmacological interventions

## Abstract

Heart failure (HF) poses a significant global health burden, necessitating a profound understanding of its multifaceted dimensions. This comprehensive review aims to unravel the etiology, decode pathophysiological mechanisms, navigate diagnostic modalities, explore pharmacological interventions, advocate lifestyle modifications, and chart the horizon of emerging therapies in the complex landscape of chronic cardiac dysfunction. The exploration of HF begins with an insightful journey into its diverse etiological factors, encompassing genetic predispositions, hypertension, and coronary artery disease. Delving into pathophysiological mechanisms, this review elucidates the intricate processes of cardiac remodeling, neurohormonal activation, and cellular dysfunction that underlie the progression of HF. Diagnostic modalities play a pivotal role in unraveling the mysteries of HF by examining advanced imaging techniques, biomarkers, and comprehensive clinical assessments. The pharmacological interventions section provides an in-depth analysis of traditional medications, such as diuretics and angiotensin-converting enzyme inhibitors, while highlighting the emergence of novel drug classes transforming HF management. Advocating lifestyle modifications emphasizes the crucial role of diet, exercise, smoking cessation, and alcohol moderation in enhancing patient outcomes. Lastly, the review delves into the promising horizon of emerging therapies, offering a glimpse into current research, innovative treatment approaches, and potential breakthroughs. As HF management faces challenges in patient compliance, healthcare access, and education, this comprehensive review aims to equip healthcare professionals and researchers with a holistic understanding of chronic cardiac dysfunction’s intricacies. In conclusion, synthesizing key findings emphasizes the need for an integrated and multidimensional approach to effectively address the complex landscape of heart failure.

## 1. Introduction

Heart failure (HF) is a formidable health challenge worldwide, demanding a nuanced exploration of its multifaceted nature to inform effective clinical practices and advance research endeavors. The World Health Organization estimates that over 26 million people suffer from HF globally.^[1]^ This necessitates a comprehensive understanding of the condition, encompassing its etiology, pathophysiological mechanisms, diagnostic modalities, pharmacological interventions, lifestyle modifications, and the promising landscape of emerging therapies.

The prevalence of HF is notably influenced by various factors, including age, lifestyle, and preexisting health conditions.^[2]^ As the aging population continues to grow, coupled with an increasing prevalence of cardiovascular risk factors, the burden of HF is expected to rise substantially.^[3]^ Thus, a robust exploration of the etiological factors contributing to HF is imperative for effective prevention and management strategies.

This review embarks on a journey into the intricate web of HF etiology, aiming to shed light on genetic predispositions, hypertension, and coronary artery disease (CAD) as prominent contributors.^[4–6]^ By unraveling the complex interplay of these factors, we can deepen our understanding of why and how HF manifests, paving the way for targeted interventions and preventive measures. Through this exploration, we aim to provide a foundation for subsequent sections that delve into the pathophysiological underpinnings of HF and strategies for accurate diagnosis and effective intervention.

## 2. Objective of study

The primary objective of this comprehensive review is to consolidate and analyze the existing knowledge surrounding HF, explicitly unraveling its etiology, decoding pathophysiological mechanisms, navigating diagnostic modalities, exploring pharmacological interventions, advocating lifestyle modifications, and charting the horizon of emerging therapies. By systematically examining each facet of HF, we aim to provide healthcare professionals, researchers, and policymakers with a nuanced understanding of the complexities associated with chronic cardiac dysfunction.

Through an exploration of the etiological factors, we seek to highlight the intricate web of genetic predispositions, hypertension, and CAD, contributing to a holistic comprehension of why individuals may succumb to HF.^[4–6]^ Building upon this foundation, our objective includes a detailed analysis of the pathophysiological mechanisms driving the progression of HF, including cardiac remodeling, neurohormonal activation, and cellular dysfunction.

The study further aims to evaluate the various diagnostic modalities available for HF, including advanced imaging techniques, biomarkers, and comprehensive clinical assessments, emphasizing the importance of accurate and timely diagnosis for effective management.^[7–9]^ Pharmacological interventions, a cornerstone of HF management, will be critically examined, encompassing traditional medications such as diuretics, ACE inhibitors, and newer drug classes transforming the treatment landscape.^[10,11]^

Advocating lifestyle modifications is another crucial objective, exploring the impact of diet, exercise, smoking cessation, and alcohol moderation on improving patient outcomes and overall quality of life (QoL).^[12–14]^ Lastly, we aim to provide insights into the horizon of emerging therapies, offering a glimpse into current research, innovative treatment approaches, and potential breakthroughs that may shape the future of HF management.

This study aims to synthesize and disseminate comprehensive knowledge that can inform clinical decision-making, inspire further research initiatives, and contribute to the ongoing efforts to address the global burden of HF effectively.

## 3. Literature review

### 3.1. Definition and epidemiology

HF is a complex clinical syndrome characterized by the heart’s inability to pump blood effectively to meet the body’s metabolic demands.^[15]^ This condition manifests when the heart’s pumping capacity is compromised, leading to symptoms such as shortness of breath, fatigue, and fluid retention.^[16]^ Understanding the definition is crucial for precise diagnosis and targeted management strategies.

The epidemiology of HF underscores its significant impact on public health. Globally, HF affects over 26 million people, with an estimated prevalence of 1% to 2% of the adult population.^[1]^ The incidence and prevalence of HF increase with age, making it a predominant health concern in the elderly population.^[17]^ As the aging demographic expands and the prevalence of cardiovascular risk factors rises, the burden of HF is anticipated to escalate in the coming years.^[3]^

Geographical and socioeconomic variations also play a role in HF epidemiology, with higher rates observed in specific regions and among populations with limited access to healthcare.^[18]^ Understanding these epidemiological patterns is essential for developing targeted prevention and intervention strategies.

### 3.2. Etiology of HF

HF is a multifactorial condition influenced by various etiological factors that contribute to its development. Understanding these factors is crucial for tailoring effective preventive and therapeutic strategies.

1. **Genetic predispositions^[4]^**:
  - A family history of HF can significantly increase the risk of developing the condition.
  - Genetic mutations affecting cardiac function and structure play a role in some cases.
2. **Hypertension^[5]^**:
  - Elevated blood pressure over time can lead to structural changes in the heart, impairing its ability to pump effectively.
  - Hypertension is a significant risk factor for the development of HF.
3. **CAD^[6]^**:
  - Atherosclerosis and ischemic heart disease can result in myocardial infarction, leading to impaired cardiac function.
  - CAD is a leading cause of HF, emphasizing the importance of cardiovascular health.
4. **Valvular heart disease^[19]^**:
  - Malfunctioning heart valves can disrupt normal blood flow, placing strain on the heart.
  - Valve diseases, such as aortic stenosis or mitral regurgitation, contribute to HF development.
5. **Myocarditis and cardiomyopathies^[20]^**:
  - Inflammation of the heart muscle (myocarditis) and various cardiomyopathies can compromise heart function.
  - Dilated, hypertrophic, or restrictive cardiomyopathies contribute to different forms of HF.
6. **Diabetes mellitus^[21]^**:
  - Diabetes is a metabolic condition that increases the risk of cardiovascular complications, including HF.
  - Insulin resistance and hyperglycemia contribute to structural and functional changes in the heart.

## 4. Pathophysiological mechanisms

The progression of HF involves intricate pathophysiological mechanisms that impact the structure and function of the heart. Examining these processes is essential for developing targeted interventions and improving patient outcomes.

1. **Cardiac remodeling:**
  - Chronic stress on the heart, often due to hypertension or myocardial infarction, initiates cardiac remodeling.
  - Remodeling involves changes in the heart’s size, shape, and function, ultimately leading to impaired contractility and pump function.^[22]^
2. **Neurohormonal activation:**
  - In response to reduced cardiac output, neurohormonal systems, such as the renin-angiotensin-aldosterone system and sympathetic nervous system, are activated.^[23]^
  - Prolonged activation of these systems contributes to vasoconstriction, sodium and water retention, and increased cardiac workload, exacerbating HF.
3. **Cellular dysfunction:**
  - Impaired cellular function within myocardial cells, including calcium handling and energy production alterations, contributes to contractile dysfunction.^[24]^
  - Cellular changes involve inflammation, oxidative stress, and apoptosis, compromising the heart’s ability to function optimally.
4. **Endothelial dysfunction^[25]^**:
  - Dysfunction of the endothelium, the inner lining of blood vessels, contributes to impaired vasodilation and increased vascular resistance.
  - This endothelial dysfunction contributes to elevated afterload, placing additional strain on the heart.
5. **Mitochondrial dysfunction^[26]^**:
  - Altered mitochondrial function affects energy production in cardiac cells.
  - Impaired mitochondrial function reduces ATP availability, impacting the energy-demanding cardiac contraction and relaxation processes.

## 5. Diagnostic modalities

Accurate diagnosis is pivotal in the effective management of HF. Various diagnostic modalities offer valuable insights into the structural and functional aspects of the heart, aiding clinicians in making informed decisions regarding patient care.

1. **Imaging techniques:**
  - **Echocardiography^[27]^**:
    - Utilizes sound waves to create detailed images of the heart’s structure and function.
    - Assesses ventricular function and valvular abnormalities and detects structural changes indicative of HF.
  - **Cardiac magnetic resonance imaging^[28]^**:
    - Provides high-resolution images of the heart, aiding in assessing myocardial tissue, chambers, and blood flow.
    - Valuable for identifying ischemic and nonischemic causes of HF.
  - **Computed tomography angiography^[29]^**:
    - Offers detailed images of the coronary arteries, helping to identify blockages or stenosis.
    - It helps assess CAD as a contributing factor to HF.
2. **Biomarkers:**
  - **Natriuretic peptides (BNP and NT-proBNP)^[30]^**:
    - Elevated levels indicate myocardial stress and are indicative of HF.
    - They are used for both diagnosis and assessing the severity of HF.
  - **Troponin^[31]^**:
    - Elevated troponin levels may suggest myocardial injury, which can contribute to HF.
    - It helps in identifying acute coronary syndromes and their impact on cardiac function.
3. **Clinical assessment:**
  - **Symptomatology:**
    - Evaluation of symptoms such as dyspnea, fatigue, and edema provides essential clinical insights.
    - Symptoms are often assessed using standardized scales to quantify their impact on the patient’s QoL.^[32]^
  - **Physical examination:**
    - Signs of HF, including elevated jugular venous pressure, third heart sound (S3), and peripheral edema, aid in diagnosis.^[33]^

## 6. Pharmacological interventions

Pharmacological management is central to treating HF comprehensively. Various drug classes aim to alleviate symptoms, improve cardiac function, and modify the underlying pathophysiological processes contributing to HF progression.

1. **Diuretics^[34]^**:
  - **Loop diuretics (e.g., furosemide):**
    - Reduce fluid overload by promoting diuresis.
    - Alleviate symptoms of congestion, such as edema and shortness of breath.
2. **Angiotensin-converting enzyme (ACE) inhibitors^[35]^**:
  - **Enalapril, lisinopril:**
    - Inhibit the conversion of angiotensin I to angiotensin II, reducing vasoconstriction and aldosterone release.
    - Improve symptoms, decrease hospitalizations, and enhance survival.
3. **Beta-blockers^[36]^**:
  - **Carvedilol, metoprolol:**
    - Block the effects of sympathetic stimulation on the heart, reducing heart rate and myocardial oxygen demand.
    - Improve left ventricular function and decrease mortality.
4. **Angiotensin receptor blockers^[37]^**:
  - **Valsartan, losartan:**
    - Block the action of angiotensin II receptors, similar to ACE inhibitors.
    - Used in patients intolerant to ACE inhibitors.
5. **Mineralocorticoid receptor antagonists^[38]^**:
  - **Spironolactone, eplerenone:**
    - Block aldosterone receptors, reducing sodium and water retention.
    - Improve symptoms and decrease mortality, particularly in severe HF.
6. **Sacubitril/valsartan (ARNI)^[39]^**:
  - Combines a neprilysin inhibitor (sacubitril) with an angiotensin receptor blocker (valsartan).
  - Enhances natriuretic peptides, reducing congestion and mortality compared to ACE inhibitors alone.
7. **Ivabradine^[40]^**:
  - Inhibits the If current in the sinoatrial node, reducing heart rate without affecting contractility.
  - Used in select patients to reduce symptoms and hospitalizations.
8. **Inotropes (digoxin)^[41]^**:
  - Increases myocardial contractility and decreases heart rate.
  - Reserved for specific cases due to potential toxicity.

As the landscape of HF management evolves, newer therapies and combinations continue to emerge, providing additional options for tailoring treatment to individual patient needs. Established clinical guidelines summarize key recommendations for HF management in Table 1. However, the subsequent sections will explore lifestyle modifications that complement pharmacological interventions, offering a holistic approach to managing HF.

**Table 1:** Global guideline perspective on heart failure management.

**ACC/AHA guidelines** The ACC/AHA guidelines serve as a cornerstone in shaping evidence-based practices for preventing, diagnosing, and managing HF. These regularly updated guidelines provide comprehensive recommendations grounded in scientific evidence and expert consensus. Here, we outline key aspects covered in the ACC/AHA guidelines: **Definition and classification^[1]^**: The guidelines clearly define HF and its classification based on the severity of symptoms and structural heart disease.^[2]^ This foundational section guides clinicians in accurately diagnosing and categorizing patients with HF. **Diagnostic evaluation^[3]^**: Recommendations for diagnostic evaluations include a thorough clinical assessment, laboratory tests, imaging studies, and, if necessary, hemodynamic monitoring.^[4]^ The guidelines emphasize the importance of identifying underlying causes and contributing factors. **Pharmacological interventions^[5]^**: The ACC/AHA guidelines offer a comprehensive overview of pharmacological management, including recommendations for ACEIs, ARBs, beta-blockers, diuretics, and more.^[6]^ Specific guidance is provided for different patient populations and coexisting conditions. **Device-based therapies^[7]^**: Recommendations for device-based therapies cover ICDs, CRT, and left ventricular assist devices.^[8]^ The guidelines outline criteria for device implantation and updates on emerging technologies. **Lifestyle modifications^[9]^**: Lifestyle interventions, including dietary recommendations, exercise, and weight management, are emphasized in the guidelines.^[10]^ The importance of patient education and self-care is highlighted to enhance overall well-being. **Monitoring and follow-up^[11]^**: The ACC/AHA guidelines guide monitoring patients with HF, including regular follow-up assessments, adjustments to medications, and the incorporation of patient-reported outcomes.^[12]^ Continuous evaluation aims to optimize therapy and address changes in patient status. **Transitions of care^[13]^**: Recommendations for transitions of care cover the coordination between inpatient and outpatient settings, emphasizing the importance of communication and comprehensive discharge planning.^[14]^ Smooth transitions aim to reduce hospital readmissions and improve long-term outcomes. **Special populations and comorbidities^[15]^**: The guidelines recognize the unique considerations for special populations, such as older adults, women, and those with comorbidities.^[16]^ Tailored approaches are outlined to address the complexities associated with diverse patient groups. **Research and innovations^[17]^**: The guidelines acknowledge the dynamic nature of HF research and encourage participation in clinical trials, fostering innovation and advancements in the field.^[18]^ Emerging therapies and technologies are integrated as evidence accumulates.
**ESC guidelines** The ESC guidelines on HF management offer a European perspective and evidence-based recommendations for healthcare professionals. These guidelines cover a broad spectrum of HF prevention, diagnosis, and treatment topics. Here’s an overview of key aspects included in the ESC guidelines: **Definition and classification^[1]^**: The ESC guidelines clearly define HF and its classification based on symptoms, structural heart disease, and ejection fraction.^[2]^ The classification system helps clinicians stratify patients for appropriate management strategies. **Diagnostic approach^[3]^**: The guidelines outline a systematic diagnostic approach, including clinical assessment, laboratory tests, imaging studies, and the role of biomarkers such as natriuretic peptides.^[4]^ Recommendations emphasize the importance of accurate diagnosis and identification of potential reversible causes. **Pharmacological management^[5]^**: ESC guidelines offer detailed recommendations on pharmacological interventions, encompassing ACE inhibitors, beta-blockers, mineralocorticoid receptor antagonists, and other therapeutic agents.^[6]^ The guidelines guide selecting and titrating medications based on individual patient characteristics. **Device-based therapies^[7]^**: Device-based therapies, such as ICDs and CRT, are addressed with specific indications and considerations.^[8]^ The ESC guidelines highlight the role of devices in reducing mortality and improving outcomes. **Comprehensive lifestyle recommendations^[9]^**: Lifestyle modifications, including dietary advice, physical activity recommendations, and smoking cessation strategies, are integral to the ESC guidelines.^[10]^ A holistic approach to patient care incorporates behavioral interventions. **Patient education and shared decision-making^[11]^**: The guidelines emphasize the importance of patient education and shared decision-making in managing HF.^[12]^ Informed patients are better equipped to participate in their care plans actively. **Monitoring and follow-up^[13]^**: Regular monitoring and follow-up strategies are outlined to assess treatment response, adjust medications, and address changes in patient status.^[14]^ The guidelines stress the importance of ongoing care to optimize outcomes. **HFpEF^[15]^**: Specific recommendations for the diagnosis and management of HFpEF, a subtype of HF, are included in the guidelines.^[16]^ Strategies for addressing comorbidities in HFpEF patients are also covered. **Implementation and quality indicators^[17]^**: The ESC guidelines include guidance on implementing HF management strategies into clinical practice and quality indicators to assess care delivery.^[18]^ Monitoring and evaluating care processes contribute to improving healthcare quality.
**HFSA guidelines** The HFSA guidelines shape evidence-based practices for managing HF in the United States. These guidelines offer comprehensive recommendations for HF prevention, diagnosis, and treatment. Here’s an overview of key aspects included in the HFSA guidelines: **Definition and classification^[1]^**: The HFSA guidelines define HF and classification based on symptoms and ejection fraction, guiding clinicians in categorizing patients for appropriate management.^[2]^ The classification system aids in tailoring interventions to individual patient profiles. **Diagnostic evaluation^[3]^**: Recommendations for diagnostic evaluation encompass a thorough clinical assessment, laboratory tests, imaging studies, and the role of biomarkers such as B-type natriuretic peptide.^[4]^ The guidelines emphasize the importance of accurate diagnosis and consideration of comorbid conditions. **Pharmacological management^[5]^**: HFSA guidelines offer detailed recommendations on pharmacological interventions, including ACEIs, ARBs, beta-blockers, and aldosterone antagonists.^[6]^ Specific guidance is provided for titration and monitoring of medications. **Device-based therapies^[7]^**: Device-based therapies, such as ICDs and CRT, are covered with indications and considerations for implementation.^[8]^ The guidelines highlight the role of devices in reducing mortality and improving outcomes. **Comprehensive lifestyle recommendations^[9]^**: Lifestyle modifications, including dietary advice, physical activity recommendations, and strategies for smoking cessation, are integral components of the HFSA guidelines.^[10]^ A holistic approach to patient care includes behavioral interventions. **Management of comorbidities^[11]^**: The guidelines address the management of common comorbidities in HF patients, such as diabetes, chronic kidney disease, and hypertension.^[12]^ Comprehensive care considers the impact of comorbid conditions on HF outcomes. **Care transitions and palliative care^[13]^**: HFSA guidelines provide recommendations for smooth care transitions between inpatient and outpatient settings, along with considerations for palliative care and advanced care planning.^[14]^ Palliative care guidance emphasizes a patient-centered approach to enhance quality of life. **Special populations^[15]^**: Specific considerations for special populations, such as older adults, women, and individuals with HFpEF, are addressed in the guidelines.^[16]^ Tailored approaches consider the unique aspects of diverse patient groups. **Patient education and shared decision-making^[17]^**: The HFSA guidelines underscore the importance of patient education and shared decision-making in managing HF.^[18]^ Informed patients are better equipped to participate in their care plans actively.
**NICE guidelines** The NICE guidelines provide evidence-based recommendations for preventing, diagnosing, and managing HF in the United Kingdom. These guidelines aim to guide healthcare professionals in delivering optimal care for individuals with HF. Here’s an overview of key aspects included in the NICE guidelines: **Definition and diagnosis^[1]^**: NICE guidelines define HF and provide criteria for diagnosis, emphasizing the importance of clinical assessment, biomarkers, and imaging studies.^[2]^ The guidelines assist healthcare professionals in accurately identifying and categorizing HF patients. **Pharmacological management^[3]^**: Recommendations for pharmacological interventions include guidance on the use of ACEIs, ARBs, beta-blockers, and diuretics.^[4]^ NICE guidelines specify titration, monitoring, and considerations for individual patient profiles. **Device-based therapies^[5]^**: Device-based therapies, such as ICDs and CRT, are covered with indications and considerations for implementation.^[6]^ The guidelines highlight the role of devices in reducing mortality and improving outcomes. **Monitoring and follow-up^[7]^**: NICE guidelines recommend monitoring HF patients, including regular assessments, medication adjustments, and considerations for patient-reported outcomes.^[8]^ Ongoing evaluation aims to optimize therapy and address changes in patient status. **Lifestyle recommendations^[9]^**: Lifestyle modifications, including dietary advice, exercise, and smoking cessation, are integral to the NICE guidelines.^[10]^ A holistic approach to patient care includes behavioral interventions. **Management of comorbidities^[11]^**: The guidelines address the management of common comorbidities in HF patients, such as diabetes, chronic kidney disease, and hypertension.^[12]^ Comprehensive care considers the impact of comorbid conditions on HF outcomes. **Special populations^[13]^**: Specific considerations for special populations, such as older adults, women, and individuals with HFpEF, are included in the guidelines.^[14]^ Tailored approaches consider the unique aspects of diverse patient groups. **Patient education and shared decision-making^[15]^**: NICE guidelines underscore the importance of patient education and shared decision-making in managing HF.^[16]^ Informed patients are better equipped to participate in their care plans actively.
**WHO Guidelines on HF Management** The WHO guidelines contribute to the global perspective on HF management, offering recommendations applicable in various healthcare settings worldwide. These guidelines encompass different HF prevention, diagnosis, and treatment topics. Here’s an overview of key aspects covered in the WHO guidelines: **Preventive strategies^[1]^**: WHO guidelines include recommendations for preventive strategies, emphasizing the importance of addressing cardiovascular risk factors and promoting heart-healthy lifestyles.^[2]^ Strategies may focus on public health initiatives to reduce the incidence of HF. **Diagnostic evaluation^[3]^**: The guidelines guide the diagnostic evaluation of HF, including clinical assessment, laboratory tests, imaging studies, and biomarkers.^[4]^ Recommendations aim to facilitate accurate and accessible diagnosis, especially in resource-limited settings. **Pharmacological interventions^[5]^**: WHO guidelines offer recommendations on pharmacological interventions, considering the availability and affordability of medications in diverse healthcare settings.^[6]^ The guidelines may address essential medications for HF management. **Device-based therapies^[7]^**: Device-based therapies, such as ICDs and CRT, may be covered with a focus on feasibility and cost-effectiveness.^[8]^ The guidelines consider the global accessibility of advanced medical technologies. **Lifestyle modifications^[9]^**: Lifestyle modifications, including dietary recommendations, physical activity, and smoking cessation, are integral to the WHO guidelines.^[10]^ Strategies may focus on culturally appropriate approaches to enhance adherence. **Integrated care and health systems^[11]^**: WHO guidelines emphasize the importance of integrated care and health systems strengthening for HF management.^[12]^ Recommendations may address challenges and opportunities for improving healthcare infrastructure globally. **Patient education and community engagement^[13]^**: The guidelines underscore the significance of patient education and community engagement in HF management.^[14]^ Strategies may include health literacy initiatives and community-based programs. **Global collaboration and research^[15]^**: WHO guidelines may highlight the need for global collaboration in HF research and the importance of data collection to understand the global burden of HF.^[16]^ The guidelines may encourage participation in international research efforts.
**Clinical practice guidelines for HF in Japan** Japan’s Clinical Practice Guidelines for HF provide a unique perspective on managing HF tailored to the Japanese population and healthcare system. These guidelines offer evidence-based recommendations to guide healthcare professionals in delivering optimal care for individuals with HF. Here’s an overview of key aspects covered in the guidelines: **Definition and classification^[1]^**: The Japanese guidelines clearly define HF and its classification based on symptoms, structural heart disease, and ejection fraction.^[2]^ This classification system assists healthcare professionals in categorizing patients for appropriate management. **Diagnostic approach^[3]^**: Recommendations for the diagnostic approach cover clinical assessment, laboratory tests, imaging studies, and the use of specific biomarkers relevant to the Japanese context.^[4]^ The guidelines aim to facilitate accurate diagnosis, considering factors unique to the Japanese population. **Pharmacological management^[5]^**: Specific recommendations for pharmacological interventions, including ACEIs, ARBs, beta-blockers, and other therapeutic agents, are tailored to the Japanese population.^[6]^ The guidelines guide medication titration and monitoring. **Device-based therapies^[7]^**: Device-based therapies, such as ICDs and CRT, are covered with indications and considerations relevant to the Japanese healthcare landscape.^[8]^ The guidelines highlight the role of devices in improving outcomes for Japanese patients. **Lifestyle recommendations^[9]^**: Lifestyle modifications, including dietary advice, physical activity recommendations, and strategies for smoking cessation, are integral components of the guidelines, considering cultural and nutritional preferences in Japan.^[10]^ A holistic approach to patient care incorporates behavioral interventions. **Management of comorbidities^[11]^**: The guidelines address the management of common comorbidities in HF patients in the Japanese context, such as hypertension and diabetes.^[12]^ Comprehensive care considers the impact of comorbid conditions on HF outcomes. **Follow-up and monitoring^[13]^**: Recommendations for follow-up and monitoring aim to assess treatment response, adjust medications, and address changes in patient status relevant to the Japanese healthcare system.^[14]^ Ongoing evaluation is essential for optimizing therapy. **Patient education and shared decision-making^[15]^**: The guidelines underscore the importance of patient education and shared decision-making in managing HF in Japan.^[16]^ Informed patients are better equipped to participate in their care plans actively.

ACC = American College of Cardiology, ACEIs = angiotensin-converting enzyme inhibitors, AHA = American Heart Association, ARBs = angiotensin II receptor blockers, CRT = cardiac resynchronization therapy, ESC = European Society of Cardiology, HF = heart failure, HFpEF = heart failure with preserved ejection fraction, HFSA = Heart Failure Society of America, ICDs = implantable cardioverter-defibrillators, LVADs = left ventricular assist devices, NICE = National Institute for Health and Care Excellence, WHO = World Health Organization.

## 7. Lifestyle modifications

In conjunction with pharmacological interventions, lifestyle modifications are integral to managing HF. These changes optimize overall health, enhance well-being, and improve HF patients’ long-term stability.

1. **Diet and nutrition^[12]^**:
  - **Low-sodium diet:**
    - Restricting sodium intake helps manage fluid retention and reduce symptoms of congestion.
    - Encouraging a diet rich in fruits, vegetables, and lean proteins supports overall cardiovascular health.
  - **Heart-healthy diet:**
    - Emphasizing a diet low in saturated and trans fats supports cholesterol control and cardiovascular health.
    - The Mediterranean or DASH (Dietary Approaches to Stop Hypertension) diet is often recommended.
2. **Regular exercise^[13]^**:
  - **Cardiac rehabilitation:**
    - Structured exercise programs tailored to individual capacities improve exercise tolerance and QoL.
    - Regular physical activity contributes to overall cardiovascular health.
  - **Aerobic exercise:**
    - Moderate-intensity aerobic activities, such as walking or cycling, enhance cardiovascular fitness.
    - Exercise tolerance improves, and symptoms like shortness of breath may decrease.
3. **Smoking cessation^[14]^**:
  - **Tobacco abstinence:**
    - Smoking cessation is crucial to reducing the risk of cardiovascular events and improving overall health.
    - Support programs and pharmacotherapy aid in smoking cessation.
4. **Moderation in alcohol consumption:**
  - **Alcohol management:**
  - Moderating alcohol intake is advised, as excessive consumption can contribute to HF.
  - Patients are encouraged to adhere to recommended limits or abstain based on individual health status.
5. **Weight management:**
  - **Maintaining healthy weight:**
    - Achieving and maintaining a healthy weight reduces the workload on the heart.
    - Weight loss may be recommended for overweight or obese individuals with HF.

Lifestyle modifications empower individuals with HF to actively participate in their care actively, promoting overall well-being and improving treatment outcomes. As we navigate the complexities of HF management, the subsequent sections will explore the promising horizon of emerging therapies, shedding light on current research initiatives and innovative approaches that may shape the future of HF management.

## 8. Emerging therapies

The landscape of HF management is evolving with ongoing research and the emergence of innovative therapeutic approaches. These promising strategies offer potential breakthroughs in addressing the complexities of chronic cardiac dysfunction.

1. **Gene and cell therapies^[42]^**:
  - **Gene editing techniques:**
    - Advances in gene editing technologies hold promise for correcting genetic mutations contributing to HF.
    - Personalized gene therapies may mitigate underlying genetic factors.
  - **Stem cell therapy:**
    - Utilizing stem cells to regenerate damaged myocardium shows potential for improving cardiac function.
    - Ongoing research explores the optimal sources and delivery methods for stem cell therapy.

2. **Precision medicine^[43]^**:
  - **Biomarker-guided therapy:**
    - Tailoring treatment based on individual biomarker profiles allows for more precise and targeted interventions.
    - It is identifying specific patient subgroups that may benefit from certain medications.
3. **Immunomodulation^[44]^**:
  - **Anti-inflammatory therapies:**
    - Targeting inflammation in HF to mitigate the impact of chronic immune activation.
    - Investigational drugs aim to modulate the immune response and improve cardiac function.
4. **Device therapies^[45]^**:
  - **Implantable cardioverter-defibrillators and cardiac resynchronization therapy:**
  - Advancements in device therapies aim to optimize device programming and patient selection.
  - Improving the effectiveness of implantable cardioverter-defibrillators and cardiac resynchronization therapy devices to prevent sudden cardiac death and synchronize ventricular contractions.
5. **Artificial intelligence (AI) in healthcare^[46]^**:
  - **Predictive analytics:**
    - AI applications analyze vast datasets to predict HF exacerbations.
    - Enhancing risk stratification and facilitating timely interventions.
6. **Telemedicine and remote monitoring^[47]^**:
  - **Virtual health platforms:**
    - Telehealth and remote monitoring solutions enable continuous patient monitoring.
    - Facilitating early detection of worsening symptoms and timely adjustments to treatment plans.

As research progresses, these emerging therapies potentially revolutionize HF management, offering more targeted and personalized approaches. The subsequent sections will address the challenges in managing chronic cardiac dysfunction, including issues related to patient compliance, access to healthcare, and patient education.

## 9. Challenges in managing chronic cardiac dysfunction

Effectively addressing chronic cardiac dysfunction, such as HF, comes with several challenges that impact patient outcomes and healthcare delivery.

1. **Patient compliance:**
  - **Medication adherence:**
    - Encouraging consistent adherence to complex medication regimens can be challenging for patients with HF.
    - noncompliance may lead to worsening symptoms and increased hospitalizations.
2. **Access to healthcare:**
  - **Health disparities:**
    - Disparities in healthcare access and quality contribute to variations in HF outcomes.
    - Limited access to specialized care may result in delayed diagnosis and suboptimal management.
  - **Remote areas and rural populations:**
    - Residents in remote or rural areas may face challenges accessing specialized cardiac care.
    - Telemedicine initiatives aim to bridge this gap but are not universally accessible.
3. **Patient education:**
  - **Understanding the condition:**
    - Health literacy may help patients’ understanding of HF and its management.
    - Comprehensive patient education is crucial for fostering self-management and lifestyle modifications.
4. **Comorbidities and polypharmacy:**
  - **Multimorbidity:**
    - The presence of multiple comorbid conditions alongside HF complicates treatment.
    - Balancing the management of various conditions while avoiding medication interactions is challenging.
  - **Polypharmacy:**
    - Patients with HF often require multiple medications, increasing the risk of polypharmacy.
    - Careful medication management is necessary to prevent adverse effects and ensure optimal treatment.
5. **Cost of treatment:**
  - **Financial barriers:**
    - The cost of medications, devices, and specialized care may pose financial challenges for patients.
    - High out-of-pocket expenses may deter individuals from seeking necessary healthcare.
6. **Transition of care:**
  - **Hospital to home transition:**
    - Ensuring a smooth transition from hospital care to home prevents readmissions.
    - Coordination between healthcare providers and clear communication with patients is essential.
7. **Advanced care planning:**
  - **End-of-life care discussions:**
    - Discussing advanced care planning, including end-of-life preferences, can be emotionally challenging.
    - Initiating these conversations is crucial for aligning medical interventions with patients’ values and preferences.

Addressing these challenges requires a multidisciplinary approach involving healthcare providers, patients, caregivers, and policymakers. Overcoming these hurdles contributes to improved patient outcomes and a more effective response to the complex landscape of chronic cardiac dysfunction.

## 10. Economic Impact of HF

The economic burden of HF is substantial, encompassing direct healthcare costs, indirect costs related to productivity loss, and the overall impact on the economy. Understanding the economic dimensions of HF is crucial for healthcare planning, resource allocation, and developing cost-effective interventions.

1. **Direct healthcare costs:**
  - **Hospitalizations:**
    - HF is a leading cause of hospital admissions, contributing significantly to healthcare expenditures.
    - Costs associated with inpatient care, diagnostics, and procedures contribute to the economic burden.
  - **Medications and interventions:**
    - The expense of medications, including diuretics, beta-blockers, and newer therapies, adds to the economic impact.
    - Costs associated with interventions such as device implantation and cardiac rehabilitation contribute to overall spending.
2. **Indirect costs:**
  - **Productivity loss:**
    - HF can reduce work productivity and absenteeism, impacting individuals’ economic contributions.
    - Caregivers may also experience productivity loss due to caregiving responsibilities.
  - **Disability and early retirement:**
    - Severe cases of HF may lead to disability, early retirement, and decreased earning potential.
    - The long-term economic impact includes both individual and societal ramifications.
3. **Economic impact on healthcare systems:**
  - **Resource allocation:**
    - The high prevalence of HF necessitates significant resources for diagnosis, treatment, and ongoing management.
    - Healthcare systems need help in allocating resources efficiently to address the growing burden.
  - **Preventive measures and public health initiatives:**
    - Investing in preventive measures and public health initiatives can help mitigate the economic impact.
    - Early detection, lifestyle interventions, and patient education can contribute to cost savings in the long run.
4. **Cost-effectiveness of interventions:**
  - **Evaluating treatment modalities:**
    - Assessing the cost-effectiveness of different treatment modalities is crucial for optimizing healthcare spending.
    - Comparative effectiveness research aids in identifying interventions that provide value for money.
5. **Global disparities:**
  - **Variations in economic impact:**
    - The economic impact varies across regions and is influenced by healthcare infrastructure, medication access, and socioeconomic factors.
    - Understanding these disparities is essential for tailoring global health strategies.

Addressing the economic impact of HF requires a comprehensive approach that considers both short-term healthcare costs and long-term socioeconomic consequences. By quantifying these economic dimensions, policymakers and healthcare providers can develop strategies to mitigate the financial burden on individuals and healthcare systems.

## 11. Quality of life in HF

HF exerts a profound impact on the QoL of affected individuals, influencing physical, emotional, and social well-being.^[1]^ The multifaceted nature of HF symptoms, coupled with the complexities of management, underscores the importance of understanding and addressing QoL considerations.

1. **Physical impact^[2]^**:
  - **Symptom burden:**
    - HF symptoms, including dyspnea, fatigue, and exercise intolerance, significantly affect the physical capabilities of individuals.^[3]^
    - Reduced exercise tolerance and limitations in daily activities contribute to a diminished sense of well-being.
  - **Functional impairment:**
    - Progressive HF often leads to functional impairment, impacting individuals’ ability to perform routine tasks.
    - Loss of independence and increased reliance on caregivers can further compromise physical QoL.
2. **Emotional well-being^[4]^**:
  - **Psychological distress:**
    - The chronic nature of HF, coupled with the uncertainty of the disease trajectory, can induce psychological distress.^[5]^
    - Anxiety and depression are prevalent, adversely affecting emotional QoL.
  - **Impact on mental health:**
    - Cognitive impairment, often associated with HF, adds a layer of challenge to mental health.^[6]^
    - Individuals may experience difficulties in memory, concentration, and decision-making.
3. **Social dimensions^[7]^**:
  - **Social isolation:**
    - HF can lead to social isolation, as individuals may withdraw from social activities due to fatigue or fear of symptom exacerbation.^[8]^
    - Reduced social interactions contribute to a diminished sense of connectedness.
  - **Caregiver burden^[9]^**:
    - The impact of HF extends beyond the individual to caregivers, who may experience increased stress and a decline in their own QoL.^[10]^
    - Balancing caregiving responsibilities with other life commitments is a significant challenge.
4. **Impact on overall well-being^[11]^**:
  - **Quality of life assessments:**
    - Various tools, such as the Minnesota Living with HF Questionnaire, enable quantification of the impact of HF on QoL.^[12]^
    - Routine assessments provide insights into treatment efficacy and guide interventions to improve QoL.
  - **Multidisciplinary approaches:**
    - Collaborative efforts involving healthcare providers, mental health professionals, and social support networks are crucial for enhancing QoL.^[13]^
    - Addressing physical, emotional, and social dimensions holistically contributes to a more comprehensive approach to HF management.

Understanding and addressing the nuanced aspects of QoL in HF are essential for delivering patient-centered care. By prioritizing interventions that improve clinical outcomes and the overall well-being of individuals and their caregivers, healthcare providers can significantly impact the lived experience of those affected by HF.

## 12. Global perspectives on HF management

HF poses a global health challenge, and its management varies across regions due to differences in healthcare infrastructure, cultural considerations, and socioeconomic factors. International perspectives provide valuable insights into the challenges and opportunities in addressing HF internationally.

1. **Epidemiological variations^[1]^**:
  - **Prevalence across regions:**
    - The prevalence of HF varies globally and is influenced by factors such as age, cardiovascular risk profiles, and access to healthcare.^[14]^
    - Developing regions may experience different epidemiological patterns compared to more developed areas.
  - **Regional disparities:**
    - Disparities in HF prevalence and outcomes exist between high-income and low- to middle-income countries.^[15]^
    - Understanding these variations is crucial for tailoring interventions to specific regional needs.
2. **Healthcare infrastructure^[16]^**:
  - **Access to specialized care:**
    - Disparities in healthcare infrastructure impact the ability of individuals in different regions to access specialized cardiac care.^[17]^
    - Rural and underserved areas may face challenges in delivering optimal HF management.
  - **Availability of medications and technologies:**
    - Discrepancies in the availability of medications and advanced technologies influence treatment options.^[18]^
    - Access to newer therapies and diagnostic modalities may be limited in certain regions.
3. **Cultural considerations^[19]^**:
  - **Perceptions of illness:**
    - Cultural beliefs and illness perceptions influence how individuals and communities approach HF management.^[20]^
    - These cultural factors may impact adherence to treatment plans and engagement in preventive measures.
  - **Health-seeking behavior:**
    - Differences in health-seeking behavior and preferences for traditional or alternative medicine may shape HF management strategies.^[21]^
    - Tailoring interventions to align with cultural practices enhances acceptability and effectiveness.
4. **Economic implications^[22]^**:
  - **Financial barriers:**
    - Economic factors significantly impact the ability of individuals to afford HF medications and interventions.^[23]^
    - High out-of-pocket costs may hinder access to optimal care.
  - **Cost-effectiveness of interventions:**
    - Assessing the cost-effectiveness of different interventions is essential in resource-limited settings.^[24]^
    - Prioritizing interventions with the most significant impact on outcomes and cost-effectiveness is crucial.
5. **Global collaborations and initiatives^[25]^**:
  - **International research collaborations:**
    - Collaborative research initiatives facilitate the exchange of knowledge and best practices in HF management.^[26]^
    - Multinational studies contribute to a better understanding of global variations in HF.
  - **Global health policies:**
    - International organizations and policymakers shape global health policies related to HF prevention and management.^[27]^
    - Advocacy for equitable access to healthcare is essential in addressing global disparities.

Understanding the global landscape of HF management allows for developing context-specific strategies and interventions considering the diverse needs and challenges individuals and healthcare systems worldwide face. By fostering international collaborations and sharing best practices, the global community can work towards more effective and inclusive approaches to HF care.

## 13. Patient advocacy and support groups in HF management

Patient advocacy organizations and support groups play a pivotal role in empowering individuals affected by HF and their caregivers. These entities contribute to raising awareness, providing resources, and fostering a sense of community, ultimately enhancing the overall experience of those navigating the complexities of HF.

1. **Raising awareness^[1]^**:
  - **Educational campaigns:**
    - Advocacy organizations often initiate educational campaigns to raise awareness about HF, its risk factors, and preventive measures.^[2]^
    - These efforts aim to promote early detection and proactive management.
  - **Community outreach:**
    - Support groups engage in community outreach to disseminate information about HF and provide resources to at-risk populations.^[3]^
    - Targeted awareness campaigns contribute to reducing the stigma associated with cardiovascular diseases.
2. **Providing resources and information^[4]^**:
  - **Educational materials:**
    - Patient advocacy organizations develop and distribute educational materials, brochures, and online resources to inform individuals about HF management.^[5]^
    - Accessible information empowers patients to participate in their care actively.
  - **Online platforms:**
    - Virtual support groups and online forums create a space for individuals to share experiences, exchange information, and seek guidance from peers.^[6]^
    - These platforms serve as valuable resources for those unable to attend in-person meetings.
3. **Emotional support^[7]^**:
  - **Peer-to-peer support:**
    - Support groups facilitate peer-to-peer connections, allowing individuals with HF and their caregivers to share experiences and coping strategies.^[8]^
    - Emotional support from those facing similar challenges can be particularly impactful.
  - **Counseling services:**
    - Some advocacy organizations provide counseling services or connect individuals with mental health professionals to address the psychological impact of HF.^[9]^
    - Emotional well-being is a crucial component of overall QoL.
4. **Advocacy for policy changes^[10]^**:
  - **Health policy engagement:**
    - Advocacy groups engage with policymakers to influence health policies related to HF prevention, diagnosis, and treatment.^[11]^
    - By advocating for patient-centered policies, these groups contribute to improved healthcare outcomes.
  - **Research funding advocacy:**
    - Some organizations actively advocate for increased funding for HF research to drive innovation and advancements in treatment.^[12]^
    - Supporting research initiatives contributes to the development of new therapies and interventions.
5. **Promoting patient-centered care^[13]^**:
  - **Collaboration with healthcare providers:**
    - Patient advocacy groups collaborate with healthcare providers to ensure care delivery is patient-centered and aligned with patient needs and preferences.^[14]^
    - This collaboration enhances communication and shared decision-making.
  - **Empowering patients in decision-making:**
    - Providing resources that empower patients to participate in treatment decisions actively contributes to a more patient-centered approach.^[15]^
    - Informed and engaged patients are better equipped to manage their condition effectively.

Patient advocacy organizations and support groups serve as crucial allies in the journey of individuals affected by HF, contributing to a supportive ecosystem that extends beyond clinical care. By addressing patients’ informational, emotional, and advocacy needs, these groups play an integral role in promoting holistic well-being and improving outcomes in HF management.

## 14. Technological innovations in HF management

Advancements in technology have significantly impacted the landscape of HF management, offering novel approaches to monitoring, diagnosis, and treatment. These technological innovations contribute to more personalized and efficient care, enhancing the overall QoL for individuals living with HF.

1. **Wearable devices^[1]^**:
  - **Continuous monitoring:**
    - Wearable devices, such as smartwatches and fitness trackers, enable continuous monitoring of vital signs, physical activity, and sleep patterns.^[2]^
    - Real-time data empowers patients and healthcare providers to track changes and intervene proactively.
  - **Alert systems:**
    - Some wearables incorporate alert systems that notify users and healthcare teams of deviations from baseline parameters, facilitating early intervention.^[3]^
    - Timely alerts contribute to preventing exacerbations and reducing hospitalizations.
2. **Telehealth and remote monitoring^[4]^**:
  - **Virtual consultations:**
    - Telehealth platforms facilitate virtual consultations, allowing individuals with HF to connect with healthcare providers remotely.^[5]^
    - This improves access to care, particularly for those in rural or underserved areas.
  - **Implantable devices with remote monitoring:**
    - Implantable cardiac devices, such as pacemakers and defibrillators, now feature remote monitoring capabilities.^[6]^
    - This allows healthcare providers to assess device function and detect arrhythmias without needing in-person visits.
3. **Digital health platforms^[7]^**:
  - **Mobile applications:**
    - Mobile applications tailored for HF management provide tools for symptom tracking, medication reminders, and lifestyle management.^[8]^
    - These apps empower patients to engage in self-care actively.
  - **Integration with electronic health records (EHR):**
    - Integration with EHR systems allows seamless sharing of patient-generated data with healthcare providers.^[9]^
    - This enhances care coordination and ensures a comprehensive view of the patient’s health.
4. **AI^[10]^**:
  - **Predictive analytics:**
    - AI applications analyze large datasets to predict HF exacerbations and identify patients at higher risk.^[11]^
    - Predictive analytics support timely interventions and resource allocation.
  - **Personalized treatment plans:**
    - AI algorithms can analyze individual patient data to recommend personalized treatment plans based on response patterns and genetic factors.^[12]^
    - This tailored approach improves the effectiveness of interventions.
5. **Implantable sensors^[13]^**:
  - **Pressure sensors:**
    - Implantable pressure sensors can provide real-time information about pulmonary artery pressures.^[14]^
    - This data aids in optimizing medication adjustments and guiding treatment decisions.
  - **Wireless monitoring devices:**
    - Miniaturized, wireless monitoring devices can be implanted to assess cardiac function and fluid status continuously.^[15]^
    - These devices contribute to a more comprehensive understanding of the patient’s condition.
6. **Virtual reality and augmented reality^[16]^**:
  - **Rehabilitation and education:**
    - Virtual reality and augmented reality technologies are employed for rehabilitation programs and educational modules.^[17]^
    - These immersive experiences enhance patient engagement and adherence to rehabilitation protocols.

As technology continues to evolve, integrating these innovations into HF management holds the potential to revolutionize care delivery. Healthcare providers can offer more personalized, efficient, and patient-centered approaches to managing HF by leveraging the capabilities of wearables, telehealth, AI, and implantable devices.

## 15. Government policies and initiatives in HF management

Governmental involvement is pivotal in shaping effective strategies for preventing, diagnosing, and managing HF. Policies and initiatives at the national level influence healthcare infrastructure, access to resources, and the overall well-being of individuals affected by HF.

1. **Preventive health policies^[1]^**:
  - **Public health campaigns:**
    - Governments can implement public health campaigns to raise awareness about cardiovascular risk factors, emphasizing lifestyle modifications to prevent HF.^[2]^
    - Educational initiatives contribute to reducing the incidence of HF by addressing modifiable risk factors.
  - **Screening programs:**
    - Governments may initiate screening programs to identify individuals at-risk for HF, allowing for early intervention and preventive measures.^[3]^
    - Targeted screening efforts can focus on high-risk populations, promoting timely risk factor management.
2. **Healthcare infrastructure and access^[4]^**:
  - **Specialized cardiac centers:**
    - Establishing and supporting specialized cardiac centers ensures that individuals with HF have access to comprehensive care, including diagnostic facilities and expert healthcare providers.^[5]^
    - Centralized centers enhance the coordination and delivery of specialized services.
  - **Telemedicine integration:**
    - Government initiatives can support the integration of telemedicine into mainstream healthcare, improving access to HF care in remote or underserved areas.^[6]^
    - Policies facilitating reimbursement for telehealth services contribute to its widespread adoption.
3. **Research funding and support^[7]^**:
  - **Investment in cardiovascular research:**
    - Governments can fund cardiovascular research, fostering innovation in HF diagnostics, treatments, and preventive strategies.^[8]^
    - Research initiatives contribute to advancing medical knowledge and improving patient outcomes.
  - **Clinical trials support:**
    - Policies that encourage and support participation in clinical trials facilitate developing and evaluating novel therapies for HF.^[9]^
    - Incentives for industry collaboration and research partnerships contribute to a robust research ecosystem.
4. **Health information technology policies^[10]^**:
  - **EHR adoption:**
    - Government encouragement and incentives for EHR adoption enhance data sharing and care coordination for individuals with HF.^[11]^
    - A standardized approach to health information supports continuity of care.
  - **Interoperability standards:**
    - Establishing interoperability standards ensures seamless health information exchange between healthcare systems and providers.^[12]^
    - This enhances care coordination and supports a comprehensive approach to HF management.
5. **Reimbursement policies^[13]^**:
  - **Value-based care models:**
    - Implementing value-based care models, where reimbursement is tied to patient outcomes, incentivizes healthcare providers to deliver high-quality, cost-effective HF care.^[14]^
    - These models promote patient-centered care and optimal resource utilization.
  - **Pharmacoeconomic considerations:**
    - Governments may consider pharmacoeconomic evaluations when determining reimbursement policies for HF medications and interventions.^[15]^
    - Cost-effectiveness assessments inform decision-making regarding drug formularies and treatment accessibility.
6. **Patient education and support programs^[16]^**:
  - **Government-funded education initiatives:**
    - Funding for patient education programs ensures that individuals with HF receive information about their condition, treatment options, and self-management strategies.^[17]^
    - These initiatives empower patients to participate in their care actively.
  - **Community outreach and support groups:**
    - Support for community outreach and HF support groups fosters a sense of community and provides emotional support for individuals living with HF.^[18]^
    - Government involvement strengthens the impact and reach of these initiatives.

Government policies and initiatives are integral in creating an environment conducive to effective HF management. By addressing the multifaceted aspects of prevention, access to care, research, and patient support, governments contribute significantly to improving outcomes for individuals affected by HF.

## 16. Conclusion

In unraveling the complexities of HF, this comprehensive review has navigated the intricacies of etiology, pathophysiological mechanisms, diagnostic modalities, pharmacological interventions, lifestyle modifications, and emerging therapies. Drawing insights from the American College of Cardiology, American Heart Association, European Society of Cardiology, Heart Failure Society of America, National Institute for Health and Care Excellence, World Health Organization, and Japan’s Clinical Practice Guidelines, we’ve sought a global perspective on HF management.

As we chart the course through these guidelines, it becomes evident that the landscape of HF care is dynamic, adapting to regional nuances, healthcare infrastructures, and cultural considerations. Integrating wearable technologies, telehealth, artificial intelligence, and patient advocacy groups signals a transformative era in HF management, emphasizing personalized, patient-centered care.

However, amidst these advancements, challenges persist, especially in the realm of managing chronic cardiac dysfunction. Economic implications, QoL considerations, and global perspectives pose multifaceted challenges that warrant ongoing attention and collaborative efforts.

In considering the economic impact, healthcare systems must navigate cost-effective approaches without compromising the quality of care. Understanding the profound influence on patients’ QoL is paramount, necessitating holistic care approaches that extend beyond medical interventions. Embracing a global perspective involves acknowledging diverse healthcare systems, socioeconomic factors, and cultural contexts, ensuring inclusivity in the pursuit of improved HF outcomes.

In conclusion, the journey through HF management is marked by significant strides, yet the path forward demands continued research, innovative solutions, and a steadfast commitment to patient-centered care. By synthesizing knowledge from varied guidelines and embracing emerging technologies, we lay the groundwork for a future where HF is not only managed but where individuals thrive with enhanced well-being. As we look to the horizon, collaboration among healthcare providers, researchers, policymakers, and patient advocacy groups is the compass that guides us toward a future where HF is met with effective, compassionate, and globally relevant solutions.

## Author contributions

**Conceptualization:** Chukwuka Elendu, Dependable C. Amaechi, Tochi C. Elendu, Dorcas A. Amapu, Richard Ikpegbu, Mercy Asekhauno, Erica Pius, Adediwura T. Bayo-Shodipo, Nurudeen Bello, Chibuike Oguine, Nkechinyere Dike, Tanitoluwa M. Wusu-Ejalonibu, Emmanuella E. Ozigi, Aderinsola R. Olokodana, Raphael T. Nwafor, Otite Akpovona, Temitope I. Awotoye, Mutalib O. Ozigis.

**Data curation:** Chukwuka Elendu, Dependable C. Amaechi, Tochi C. Elendu, Erica Pius, Ibirongbe Amos, Tanitoluwa M. Wusu-Ejalonibu, Grace O. Otobo, Nandir J. Gonji.

**Formal analysis:** Chukwuka Elendu, Dependable C. Amaechi, Tochi C. Elendu, Samuel O. Onyekweli, Mercy Asekhauno, Ibirongbe Amos, Temitope I. Awotoye.

**Funding acquisition:** Chukwuka Elendu, Dependable C. Amaechi, Tochi C. Elendu, Osinachi K. Okoye, Samuel O. Onyekweli, Dorcas A. Amapu, Nurudeen Bello, Nkechinyere Dike, Tanitoluwa M. Wusu-Ejalonibu, Chiagozie P. Ayabazu, Nandir J. Gonji, Omotayo S. Alabi.

**Investigation:** Chukwuka Elendu, Dependable C. Amaechi, Tochi C. Elendu, Osinachi K. Okoye, Chidera A. Okezie-Okoye, Chibuike Oguine, Promise Edochie, Ibirongbe Amos, Grace O. Otobo, Aderinsola R. Olokodana, Mutalib O. Ozigis.

**Methodology:** Chukwuka Elendu, Dependable C. Amaechi, Tochi C. Elendu, Osinachi K. Okoye, Samuel O. Onyekweli, Dorcas A. Amapu, Richard Ikpegbu, Adediwura T. Bayo-Shodipo, Chidera A. Okezie-Okoye, Raphael T. Nwafor, Temitope I. Awotoye, Oluwatosin Afolabi, Omotayo S. Alabi, Mololuwa Adebayo.

**Project administration:** Chukwuka Elendu, Dependable C. Amaechi, Tochi C. Elendu, Border-ere Fiemotonghan, Richard Ikpegbu, Erica Pius, Promise Edochie, Aderinsola R. Olokodana, Chiagozie P. Ayabazu, Nandir J. Gonji, Otite Akpovona.

**Resources:** Chukwuka Elendu, Dependable C. Amaechi, Tochi C. Elendu, Dorcas A. Amapu, Erica Pius, Adediwura T. Bayo-Shodipo, Chidera A. Okezie-Okoye, Nurudeen Bello, Chibuike Oguine, Nkechinyere Dike, Joan Asekhauno, Raphael T. Nwafor, Mololuwa Adebayo.

**Software:** Chukwuka Elendu, Dependable C. Amaechi, Tochi C. Elendu, Mercy Asekhauno, Chidera A. Okezie-Okoye, Promise Edochie, Joan Asekhauno, Otite Akpovona, Temitope I. Awotoye, Oluwatosin Afolabi.

**Supervision:** Chukwuka Elendu, Dependable C. Amaechi, Tochi C. Elendu, Border-ere Fiemotonghan, Chigozirim M. Agu-ben, Adediwura T. Bayo-Shodipo, Emmanuella E. Ozigi, Grace O. Otobo, Chiagozie P. Ayabazu, Mutalib O. Ozigis.

**Validation:** Chukwuka Elendu, Dependable C. Amaechi, Tochi C. Elendu, Chigozirim M. Agu-ben, Dorcas A. Amapu, Joan Asekhauno, Omotayo S. Alabi, Mololuwa Adebayo.

**Visualization:** Chukwuka Elendu, Dependable C. Amaechi, Tochi C. Elendu, Mercy Asekhauno, Nkechinyere Dike, Raphael T. Nwafor.

**Writing – original draft:** Chukwuka Elendu, Dependable C. Amaechi, Tochi C. Elendu, Oluwatosin Afolabi.

**Writing – review & editing:** Chukwuka Elendu, Dependable C. Amaechi, Tochi C. Elendu, Border-ere Fiemotonghan, Chigozirim M. Agu-ben, Emmanuella E. Ozigi.
